# The Implementation of an Innovative Hydration Monitoring App in Care Home Settings: A Qualitative Study

**DOI:** 10.2196/mhealth.9892

**Published:** 2019-01-29

**Authors:** Alison Steven, Gemma Wilson, Lesley Young-Murphy

**Affiliations:** 1 Department of Nursing, Midwifery and Health Faculty of Health and Life Sciences Northumbria University Newcastle Newcastle upon Tyne United Kingdom; 2 NHS North Tyneside Clinical Commissioning Group North Shields United Kingdom

**Keywords:** education, frail elderly, mobile apps, patient safety, residential facilities, water-electrolyte balance

## Abstract

**Background:**

In response to marked concern regarding inadequate fluid intake recording in care homes, an innovative mobile hydration app was collaboratively developed. “Hydr8” aimed to facilitate accurate recording and communication of residents’ fluid intake and ultimately increase care quality and patient safety.

**Objective:**

The aim of this study was to examine the implementation of Hydr8 in a sample of care homes in one area in England.

**Methods:**

The principles of Realist Evaluation and Action research were drawn upon throughout the study. Overall, 5 care homes participated in this study, 3 interview-only sites and 2 case-study sites, where interviews and observations were conducted at 3 time-points. Furthermore, 28 staff members participated, including care staff, management, a registered nurse, and administrative staff.

**Results:**

Findings suggest that Hydr8 benefits practice, enhancing the understanding of hydration and person-centered care and improving staff communication. However, technical glitches hindered the seamless embedding of Hydr8 into everyday practice, and enthusiasm for long-term use was dependent on the resolution of issues. In addition, Hydr8 heightened perceptions of personal accountability, and while managers viewed this as positive, some staff members were apprehensive. However, individuals were enthusiastic about the long-term use and potential of Hydr8.

**Conclusions:**

Utilizing the findings of this study to further develop and adapt Hydr8 indicates the long-term use of Hydr8 as promising. Although perceptions of Hydr8 were primarily positive, setbacks in its implementation and use created difficulties in normalizing the solution into everyday practice. This study highlights the need for education related to hydration practice and a change of infrastructure in care home settings to implement technical solutions and changes to care.

## Introduction

### Background

Hydration management is recognized as essential to older adults’ care, with age-related variations increasing vulnerability to dehydration risk [1]. Dehydration in older adults is a patient safety concern and clinically associated with stroke, diabetes, influenza, constipation, respiratory infection, gastroenteritis, urinary tract infection, delirium, seizure, risk of falling, and mortality [1-3]. A UK-based study found over a third of older adults dehydrated on hospital admission [4].

Despite dehydration being largely preventable, care homes reportedly fail to consistently provide adequate fluids to residents [5,6]. From data obtained under freedom of information laws, it was found that 1158 care home residents in the United Kingdom suffered dehydration-related deaths between 2003 and 2012 [7]. In an analysis of death certificates, it was reported that dehydration was either the leading cause of death or a contributory factor [7].

Fundamental issues affecting hydration management are the recording of information and encouraging fluid intake. Charts for recording hydration elements such as fluid intake, fluid output, or fluid balance are frequently used with the aim of capturing fluid status and assisting deficit identification. The accurate recording of fluid-balance information is fundamental to safe care [8]; however, while monitoring fluid balance may be viewed as a simple task, completion of records is notoriously inadequate or inaccurate [9,10]. Research investigating the completion of fluid-balance charts in hospital wards found none were completed appropriately [11]. Staff shortages, lack of training, and lack of time were cited as reasons for incomplete and inaccurate charts [11]. In addition, further research highlighted problems with fluid-balance records due to a lack of communication between a hospital ward health care team and a lack of awareness and education of the importance of fluid status, especially among staff members most often completing records [8].

While hospitals have similar basic features across the globe, a care home in the United Kingdom is a residential setting in which older adults typically live in single rooms with on-site care services [12]. Care can either be paid for personally or by either the National Health Service or the local government. Care home staff requirements are regulated as part of the Health and Social Care Act 2008 and comprise largely of “care” assistants, professionally qualified nurses, and management staff.

### The Hydration Solution App

An innovative mobile hydration app “Hydr8” was developed in response to concerns regarding older adults’ hydration management and poor completion of records. Issues with the numeracy skills of some care home staff members, together with nonstandardization of recording the cup or vessel size were also considered during the coproduction of the app. While many hydration apps exist in the general market, these tend to target individuals inputting their own hydration levels. Hydr8 is an app specifically developed to be used in a health care setting by health professionals inputting hydration data for residents in a care home. In addition, unlike many existing apps, Hydr8 enables personalization to individual needs (eg, safety requirements such as thickened fluids) and preferences (eg, residents’ likes and dislikes). Furthermore, a clinical commissioning group (CCG), software development company, and care home managers worked collaboratively to coproduce Hydr8. During the development phase, a focus group approach was used to involve patients, relatives, and care home staff. This approach enabled discussion and contributions to be made regarding the appearance of the app after which a hard copy of the initial design was taken back to care homes for further comments by other staff members, patients, and relatives.

As discussed, inaccurate recording of hydration information has various implications on patients’ safety. Hydr8 aims to (1) facilitate accurate recording and communication of residents’ fluid intake; (2) automate fluid recordings and maximize the use of accessible technology; (3) enable care home staff to see cumulative totals for each resident’s intake; (4) be time-efficient, thus, releasing staff to engage in more care and leadership; (5) enable individualized care; and (6) improve awareness of the importance of hydration. Personalization to individual needs (eg, safety requirements such as thickened fluids) and preferences (eg, residents’ likes and dislikes) also increases the likelihood of maintaining hydration.

To ensure appropriate individual targets, volumes were calculated per the existing CCG and care home policy. This involved a base calculation of 30 mL fluid per kg body weight, with the addendum of 1500 mL per day as a minimum for older people [13]. This base calculation was then tailored to individually assessed needs through discussions with medical staff (ie, general practitioner or medical consultant) and other clinical staff (eg, registered nurses, dietitians, or allied health professionals involved in the individuals care). These discussions took into consideration individual health conditions, comorbidities, and treatment regimes.

**Figure 1:**
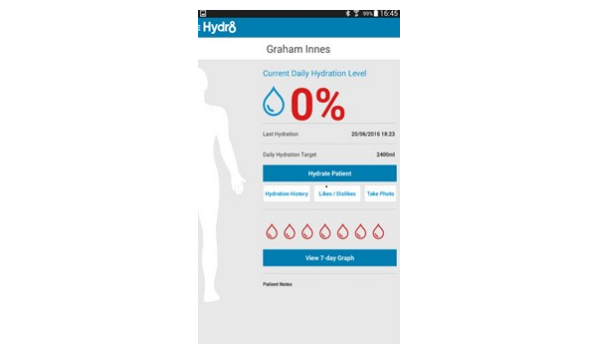
A screenshot from Hydr8 showing fluid intake information. Source: "Hydr8" Brochure produced by Elaros, North Tyneside Clinical Commissioning Group and the Academic Health Science Network North East and North Cumbria.

**Figure 2:**
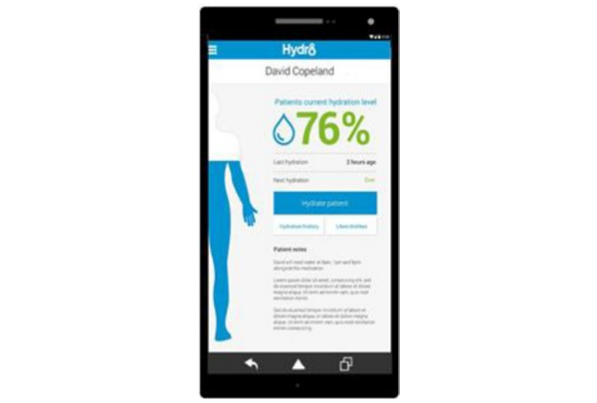
A screenshot from Hydr8 showing input screens. Source: "Hydr8" Brochure produced by Elaros, North Tyneside Clinical Commissioning Group and the Academic Health Science Network North East and North Cumbria.

Hydr8 comprises two core parts: the back-system accessed through a Web browser and a tablet-based app. Both components are accessed through username and password. The back-system permits users to add or remove residents from the app and allows them to view data across various time periods. In addition, the back-system provides opportunities for health professionals, including doctors (eg, general practitioners) or registered nurses, to access this information in real time while off-site. The app displays personalized breakdowns of fluid intake including the current daily level, last time fluids were given, and an overview of fluid intake covering the previous 7 days. These factors are visually illustrated through a body outline that fills with water as recorded fluid intake increases, with adequacy levels indicated in red, amber, and green (Figure 1). These colored levels (daily and 7-day levels; Figures 1 and 2) act as a visual signal and warning to staff.

Hydr8 enables further personalization by allowing the input of residents’ photograph, their likes and dislikes, and information on choking hazards, which is displayed using a pop-up notification. Hydr8 sends an alert when residents fall below optimum levels of fluid intake.

We aimed to explore and evaluate the pilot implementation of Hydr8 in care homes, with a particular focus on the operationalization of the system, impact on care provision, and the development needs of staff.

## Methods

### Study Design

Researching the implementation and impact of new practices or interventions in health care is problematic given the complex, context-bound nature of everyday care [14-16]. Due to the multitude of things that can influence variations in practice, traditional quantitative methods are not adequate to discern and understand the impact of complex interventions and new initiatives, be they educational or technological [16]. This difficulty is viewed as analogous to that encountered in the evaluation of complex interventions [17-19]. This inquiry, therefore, drew on the principles of Realistic Evaluation [16], which emphasizes the role of context, taking into account, for example, differing organizational settings, workforce, teams, and sociopolitical issues [20]. Akin to action research [21], interim findings were feedback to the CCG and app developers on a frequent basis to continuously develop and improve Hydr8. In addition, the Normalization Process Theory was used as a lens through which to explore the embedding and “normalizing” of Hydr8 into everyday practice. A qualitative design was utilized, encompassing observations and interviews.

### Ethical Considerations

This project was approved by the Faculty of Health and Life Sciences Research Ethics Committee at Northumbria University.

### Study Population

Data were gathered from care home sites within one CCG locality in the North of England. In this study, 5 care homes participated: 3 interview-only sites and 2 case-study sites. A sixth site declined to participate. Data collection at interview-only sites consisted of semistructured interviews at one time-point, whereas at case-study sites, it consisted of observations and semistructured interviews at 3 separate time-points.

In the study locality, the care staff age profile ranged from 19 to 60 years. Currently, for employment, UK care home staff are required to have a minimum of “National Care Certificate” qualification [22] or are obliged to work toward this within the first 6 months of employment. Depending on their role and length of time in employment, existing staff may hold levels 2-4 of the previously used “National Vocational Qualifications,” or more recent Qualifications and Credit Framework, level 2 Diploma in Care. From January 1, 2018, these previous qualifications were replaced by the “Regulated Qualifications Framework” [23].

### Data Collection

Once care home area management had given written consent for each home to be approached, GW and AS met with local management at each site to provide a study overview, discuss the study process, and disseminate information to staff. Participant information sheets and reply slips were left in a communal area of each home. If staff members were happy to participate, they were asked to leave a reply slip containing their details in a sealed box provided. This ensured anonymity of responses. Returned reply slips were collected after 7 days, and a time was arranged to return and collect data.

Semistructured interviews were conducted with staff in a quiet location in the care home. Before interviews began, participants were encouraged to ask questions about the study and then sign a consent form. Participants were advised they could withdraw from the study at any point. Interviews explored the use of the system in everyday practice, its ease and relevance, perceptions of purpose, worth, value and impact, and perceptions of development needs (Textbox 1).

In case-study sites, observations were also conducted at 3 time-points: around 1 month, 5 months, and 8 months after using the app. This allowed a continued examination of its use and changes in use over the study period. GW and AS observed and took notes quietly in the corner of a room, watching Hydr8 being used. Observations lasted up to an hour and focused on the use and usability of the system; the normalization of the system as part of everyday practice; visible impacts on care provision and outcomes; and potential education, development, and training needs (Textbox 2).

Some staff also made spontaneous comments that were recorded. Only the staff that had provided informed, written consent were observed. Semistructured interviews were undertaken at 3 time-points following the approach used at interview-only sites but with the addition of questions regarding the observations.

### Data Analysis

All interviews were recorded digitally and transcribed verbatim. Observation field notes and interview transcripts were analyzed first by each member of the research team using thematic analysis [24] and facilitated by NVIVO 10 software (QSR International Pty Ltd). The thematic analysis aims to extract themes and subthemes from interview data highlighting patterns within the dataset [24]. Specifically, the analysis followed the 6 steps of conducting the thematic analysis: familiarizing yourself with the data, generating initial codes, searching for themes, reviewing themes, defining and naming themes, and producing the report [24]. Data and initial coding were compared and discussed with the wider research team to challenge, refine, and confirm emerging findings and ensure they were rooted in the original data. In line with action research principles [21], interim findings were intermittently fed back to the development team. Realist evaluation [16] and the normalization process theory [14] were drawn upon throughout.

**Interview schedule.**

Introduction

- Seek verbal consent, answer any questions, and explain recording device

**Opening:** Prompts provided and examples sought throughout

- Have you worked here long?
- What’s your job role?
- Do you have a lot of input with the residents?
- With what they eat or drink?
- Are you involved in recording what they drink or eat?

**The Hydr8 system:** Prompts provided and examples sought throughout

- Are you aware of the new Hydr8 system?
- Do you use it? or Does everyone know about it? or Who uses it?
- Can you tell me a bit about your experiences of using it?
- What was it like to use the first time? or Did it take time to get used to?
- Were you shown how to use it? or How was it to learn to use?
- What happened if you got stuck?
- What is it like to carry about?

**Perceived impact on care provision and outcomes:** Prompts provided and examples sought throughout

- Do you use the app instead of other monitoring tools, or as a duplicate?
- How does it fit in with other tasks or practice?
- Does it make a difference? Has it changed anything?
- To your work
- To the residents
- To other staff
- Has anything changed since you started using it?

**Embedding:** Prompts provided and examples sought throughout

- What do you think about it?
- Is it relevant to your job?
- Do you see a point to it?
- What did everyone think about it?

**Observation sheet.**

**The use and usability of the system**

How long does it take to fill in?

Do they fill it in when they give drink or when drink is finished?

Do they fill it in easily?

Any technical difficulties presented with device or app?

Any usability issues with device or app?

Any verbal or visible frustration with the device or app?

Do different members of staff use the app differently?

Are they filled in enough or correctly?

Any obvious facilitators or barriers?

**Normalization of the system as part of everyday practice**

Who uses the app?

Complete every time? More than one entry per time?

Do they use in addition to other balance charts?

Do staff automatically record information on app or is it a second thought?

Any obvious facilitators or barriers?

Does the completion of the app seem to work well with other tasks or does it get in the way?

**Impacts on care provision and outcomes**

Discuss app or hydration with resident when completing?

Does it seem to affect the amount of fluid given to residents?

Do members of staff ask resident questions about adequate or inadequate hydration levels over the week?

Used differently with any residents?

Are drinks given appropriately?

Potential education, development, and training needs of staff

Any discussions about the app between staff or staff and residents or residents?

Help give by staff to other staff completing this?

Do staff or residents seem to understand its importance?

## Results

### Data Collection Statistics

In total, 10 interviews (3 at site 1, 2 at site 2, and 5 at site 3) were conducted at interview-only sites. Table 1 shows data collection for the case-study sites (n=2). Observations provided contextual understanding that helped situate and make sense of interview findings (Table 1).

Overall, 28 participants took part in interviews over 5 sites. Of these, 21 were care staff members, 5 from management, 1 administrative assistant, and 1 registered nurse. Care staff members were most frequently interviewed, as they were the staff predominantly tasked with monitoring hydration and, thus, mainly used Hydr8.

### Findings

Four interrelated themes emerged: knowledge of hydration, fitting into established systems of care, surveillance, and future gazing.

#### Knowledge of Hydration

A positive outcome of Hydr8 was the impact on care home staffs’ knowledge and understanding of hydration. Evidently, visual illustrations displaying fluid intake were more meaningful than paper-based charts.

It means less on paper

P006/care assistant

If you’re looking on the app you can think “oh-well actually…he didn’t drink that one, he could do with a bit more.” So you are pushing fluids with that particular person […] you wouldn’t if it was on paper because you wouldn’t realise, but now that it is visual, giving you the push

P025/care assistant

**Table 1:** Number of observations and interviews conducted over the 3 data collection points for the case-study sites.

Case-study sites		Visits (n)			
		Visit 1	Visit 2	Visit 3	Total
**Site 1**					
	Observations conducted	1	0	0	1
	Interviews conducted	4	2	3	9
**Site 2**					
	Observations conducted	1	0	0	1
	Interviews conducted	4	3	2	9

Through using the app, staff also gained a heightened awareness of individual preferences and individual differences in fluid intake.

You can put in their likes and dislikes if, like, they would prefer a drink. So there’s like some ladies that just like a cup of tea or milk. There’s others who, like, quite prefer a colder drink. So, it knows.

P001/care assistant

It calculates, everyone’s difference. Like, weight, size, and how much they sort of, should, need.

P001/care assistant

In addition, there were recognized changes in practice due to heighted awareness of the importance of contextual factors and individual differences, increasing person-centered care.

If it is warm, obviously, the staff are aware and I’ve heard them say, “it’s warm today, we’ll get some extra drinks out.” Or juice as opposed to a cup of tea

P003/management

The carers are a bit more involved…it’s down to height and weight, medical history […] It’s quite interesting for the carers to see that certain residents need more fluids, and other residents need less

P003/management

However, Hydr8 also had unintended consequences with some staff “frightened” (P002/care assistant) of overhydrating residents. At the time of the study, Hydr8 did not record fluid output or compute fluid balance; therefore, percentage data could show residents >100% recommended intake and, notwithstanding clarifications, this caused some anxiety. Feedback to the commissioners and developers regarding this issue prompted consideration of the future development of the app to include fluid output.

#### Fitting Into Established Systems of Care

The normalization of new technology into everyday practice is an important consideration in implementation. A number of technical issues, glitches, and knock-on effects emerged and impacted the embedding of Hydr8 into routine care. In the short-term, during the course of the study, Wi-Fi connectively was often poor, which was time-consuming for users and often resulted in the delayed record completion.

There is a lot of loading that you don’t have with paperwork […] It’s just that you can’t wait around for ages every time you want to record something

P004/care assistant

It freezes, it skips, it jumps, it doesn’t load. The Wi-Fi connection keeps coming off and doesn’t connect back up to the Wi-Fi

P011/care assistant

We use it upstairs, but the problem is through the [Wi-Fi] signal, we cannot get a signal upstairs

P020/care assistant

These faults led to time taken away from other duties and fueled staff frustration. In addition, Hydr8 repeatedly “froze,” resulting in care homes not being able to use the app for long periods of time.

There’s been a few times where it just crashes and it has been saying, unfortunately Hydr8 has stopped

P004/management

Feedback of these issues to the commissioners resulted in Wi-Fi boosters being provided to care homes with limited connectivity. While some system errors created problems, connectivity issues related to poor Wi-Fi prevailed and undoubtedly impacted the normalization of Hydr8 into daily practice. Other factors affecting normalization related to the implementation being integral to the ongoing development process. For example, during the study, most care homes (n=4) continued to complete paper-based charts to ensure no data could be lost. This duplication of information necessitated additional staff time, and many care staff members were unaware that the duplication was a short-term measure; therefore, Hydr8 was often viewed as an additional task in an already demanding workload.

I think they’ll love the app once the paperwork goes

P023/management

Furthermore, given this was a developmental phase a limited number of tablets were supplied to each care home (n=2), which was perceived as insufficient.

They haven’t necessarily been able to record at that moment in time, because somebody else has been using

P003/management

Daily routines were affected by time spent searching for devices, and participants were not always able to input data when they needed to. To manage the technical glitches, duplication, and lack of tablets, participants developed “workarounds.” Workarounds included carrying information on paper for uploading later, thus, enabling continued recording of data despite the issues experienced. These extra activities also impacted the embedding of Hydr8 into everyday working.

#### Surveillance

An interesting and unanticipated finding was participants’ perceptions that Hydr8 may function as a method of surveillance for management and external agencies. There were also apprehensions that external agencies may not fully understand data produced. Hydr8 potentially heightened accountability, and there was anxiety regarding the way in which the data were presented, how these may appear to others, and potential for increased individual accountability.

When [the external agency] come in, they do go through paperwork and bits and I don’t know how they are going to react with having to go through this

P014/care assistant

It looks like we flooded them

P011/care assistant

[The manager] can keep an eye on it as well. So, if, like, someone has missed a drink or something…he can come up straight away and say, “look, why hasn’t this one had a drink for 3, 4 hours?”

P026/care assistant

However, management staff viewed the accountability potential and the possibility to remotely access records as beneficial.

Having that accountability is important

P003/management

I’ve been over the moon with being able to observe from the office. The board of directors have actually been sitting in Harrogate observing

P016/management

I look at it from just after lunch every day. And I sit and go through it. And as soon as I see the deficit, a concern or a problem, I’m out and I want to know why

P023/management

Hydr8 was advantageous due to the possibility to view data from a 7-day period, and to do this remotely, thus, increasing the potential for communication between stakeholders.

#### Future Gazing

Respondents often talked about “technology” as a concept, and individuals often discussed the inevitability of technology becoming an integral part of their future roles.

It’s the next, sort of, generation

P001/care assistant

It’s definitely the way forward

P003/management

However, design changes such as the ability to edit inputted data and increased flexibility were repeatedly raised by participants and felt to be imperative to ensure long-term use.

They’re not editable either. I know they are on the back end, but it means that the carer makes a typing error – there’s nothing they can do

P004/management

[It needs to be] as flexible as a piece of paper

P014/care assistant

Although conditional on the elimination of technical setbacks and connectivity issues, participants were enthusiastic about the future use of Hydr8

I just think it is a brilliant idea if it all runs smoothly and works

P016/management

If it was working properly and it wasn’t getting stuck, it would be brilliant. So much easier

P026/care assistant

Furthermore, enhancements and additional functions were deemed necessary for the long-term use of Hydr8. These included “output” (P011/care assistant) and “food charts” (P023/management). Individuals also suggested the inclusion of a “24-hour personal care record” (P027/management) and additions to render Hydr8 suitable for residents with “dementia” (P023/management) or those at the “end of life stage” (P027/management). Participants felt such improvements would improve person-centered care and were enthusiastic about using Hydr8 in the future. All of these issues were feedback to the development team in a timely manner

## Discussion

### Principal Findings

Specific benefits of the Hydr8 app and solution include heightened staff understanding of hydration, increased person-centered care, and enhanced communication. However, participants also proposed additions and enhancements that would further improve Hydr8.

Hydr8 increased staff awareness and understanding of individual and contextual factors in hydration management. The importance of staff education to avoid dehydration has been highlighted in the literature [8,25-27], and systems such as Hydr8 could offer additional opportunities for work-based education relevant to the client or patient group being cared for. Information recorded using Hydr8 reflected the importance of changes in culture regarding nutrition and hydration practice and a need for a person-centered approach in recording fluid preferences and individual needs [1,27]. Understanding individual differences is an essential part of hydration management when encouraging older adults to drink more [12,27], and it was apparent that Hydr8 data were more meaningful and individual compared with traditional paper records. Indeed, the visual “KANBAN” (which means signboard or billboard in Japanese) [28] type signal given by the body shape and red, amber, or green (RAG) app display appeared to heighten staff awareness. The additional “backroom” facility, allowing managers to see the RAG rating at a glance, provides further overarching assurance, and the use of these levels of visual alert together with staff and manager monitoring may offer a certain level of “mistake proofing.” However, one unintended consequence, linked to the recording of fluid intake only, was increased anxiety felt by some staff regarding overhydration. This indicates the need for future developments of Hydr8 to include output and fluid-balance calculation and further preparation and education for staff.

Hydr8 enabled fluid intake information to be communicated more effectively given multiple individuals (with permission) could view data charts covering a 7-day period and could do so remotely. Management valued this function, as it improved their longitudinal awareness of fluid intake. Hydration could be charted over days, allowing greater sensitivity to gradual dehydration, thereby adding a further quality and safety check into the care system. Indeed, in a recent literature review, Oates and Price [27] concluded that hydration should be a collective responsibility and management also noted the increased staff accountability Hydr8 offered. Hydr8 aimed to be efficient and release staff to engage in more care and leadership activities; however, technical and implementation difficulties increased time spent recording fluid intake. One disadvantage of paper-based fluid-balance charts is that input can be time-consuming [8]; therefore, it was imperative that Hydr8 be time-efficient to make it a more “attractive” option and engender “buy-in.”

In this study, the Hydr8 system did not appear to become completely routinized or “normalized” into daily practice [26]. There was some coherence in the understanding of the goals and aims of Hydr8 and some “buy-in” by staff (illustrated by the future gazing and knowledge enhancement). However, some participants were apprehensive, perceiving Hydr8 as a potential staff surveillance and monitoring tool; this unease was heightened by the technical difficulties that resulted in recording inaccuracies. These apprehensions and staff not being fully aware of the iterative, developmental nature of the “pilot” implementation project may have limited the buy-in (or complete cognitive participation) by staff [26,29]. Furthermore, the “fit” of the Hydr8 system into existing skill sets and working practices (collective action) was hampered by the technical difficulties experienced, which disrupted the use of the app [26,29]. Despite the introduction of Wi-Fi boosters into some care homes, technical difficulties persisted because of poor Wi-Fi connectivity. With further development, these issues can be resolved, and the use of Hydr8 may result in time savings and staff being freed up for other duties. Furthermore, from this study, the importance of collaborating with software developers and companies who have an insight into, and understanding of, the complexities of the health and social care sector has emerged. This, however, remains a hypothesis and the implementation of new working practices does not always follow a preconceived logic [15]; therefore, further research is necessary to ascertain the consequences, intended or unintended, of the use of a refined Hydr8 system.

The findings illustrate the importance of technology being embedded in practice routines and culture. The implementation of technology is not simply about the device itself but the many connected sociomaterial “things” being introduced into existing social practices [30]. Introducing a new practice that is not sufficiently refined or tested may result in participants disengaging or expressing unfavorable opinions, as in this study. However, it could be argued that new practices (systems or technology) cannot be comprehensively developed before some level of implementation takes place, be it through small-scale implementation and/or piloting. Indeed, it is this period of testing and trying out that allows unforeseen issues and consequences to emerge and be resolved. Thus, the issue here was not the “piloting” in and of itself, but the need, perhaps, for much greater engagement of the care staff in the cocreation of Hydr8. Greater collaborative engagement of this section of stakeholders may have resulted in them being much more alert to emerging issues and may have raised their tolerance and allowed them to develop more complete cognitive participation, to see beyond the short-term disadvantages, specifically the duplication of information, limited tablets, and Wi-Fi connectivity issues.

This study mirrored aspects of action research [21] by investigating the implementation while also feeding back into the developmental process. As this inquiry was undertaken and resulting from the feedback of findings, commissioners and developers are working on developing this app by adding further elements, such as fluid output and nutrition, all into one app. Although there are only a small number of residents in which urine output is accurately measured in nursing homes (and during the study this was recorded for residents using traditional charts), clearly this is important in other settings and as part of the future development, adoption, and spread of the Hydr8 system.

### Limitations

One limitation of this study is that the sample was small and restricted to a specific geographical area; therefore, it is not representative of the wider population, and care must be taken when extrapolating the findings. Although not generalizable, these findings have some transferability [31]. Technical issues reduced the use of Hydr8 during this pilot study, limiting its use and preventing observations as part of data collection; however, this in itself was an important aspect of the developmental enquiry. The technical issues negatively impacted the use and effectiveness of the Hydr8 app, and technical functionality is necessary before further implementing the app in care homes. This study has highlighted the importance of monitoring ongoing technical issues during wider implementation and, as a result, the clinical commissioning group has engaged a third party that identified and rectified technical coding issues, which were at the heart of some of the problems encountered. Rectifying these issues will enable more seamless use of the app and transfer of data in the future.

Staff interviewed were those who volunteered to participate on the day, and this was the deciding factor in the numbers involved. In addition, some sites (n=2) only implemented the use of the Hydr8 app in specific parts of the care home, and the researchers were not aware of it until data collection took place. Thus, demographic details were not collected from the individual staff, and this is acknowledged as a limitation for inclusion in any future research.

### Future Considerations

Based on the findings of this pilot evaluation, Hydr8 will be further developed and evaluated. The focus of further study needs to encompass multiple aspects of use, including normalization into a daily routine, technical issues experienced, information needed on implementation, residents’ perceptions, and participants’ content and design suggestions.

A future longitudinal study is planned and will incorporate additional collection and analysis of long-term quality and safety outcomes. “Backroom” quantitative data regarding the aspects of app usage and individual resident recordings are constantly being collected by the system, and this has been ongoing since the initial implementation and piloting. While these data are visible to care homes and CCG, these are yet to be analyzed, and these would form part of planned longitudinal research. This will not only allow further assessment of its use but also include economic evaluation and residents’ perceptions of hydration management before and during the use of Hydr8. The development of plans and materials for staff preparation, education, and training for further roll out of the system is ongoing by the CCG. Such plans include investigation of peer-to-peer education, use of Hydr8 champions, and both Web-based and traditional, paper-based materials.

In addition, the study reported in this paper highlights the need for ongoing research into the human factors involved in the implementation and normalization of this system, including staff education regarding hydration and information technology literacy and individual perceptions and behaviors of residents and those of relatives and visitors. While this study took place in the United Kingdom, these issues regarding health and social care economies and delivery of best care to aging populations are of global concern.

### Conclusions

This developmental inquiry highlights the potential benefits of utilizing this electronic hydration monitoring solution in the care home setting. Specifically, the use of Hydr8 increased understanding of hydration practice and improved communication of fluid intake data; furthermore, individuals were enthusiastic about its future use in the care home settings. The developmental process led to issues being highlighted and changes being implemented during the process. However, further considerations need to be taken into account for future implementation, namely, design and technical difficulties and staff education in the care home setting. Hydr8, with the necessary amendments highlighted in this study, has the potential to effectively improve the quality and safety of care.

## Abbreviations

CCG: clinical commissioning group
RAG: red, amber, or green
